# Validation of the Arabic eHealth literacy questionnaire: a factor and Rasch analysis study

**DOI:** 10.3389/fpubh.2025.1542477

**Published:** 2025-02-07

**Authors:** Walid Al-Qerem, Osama N. Fadhil, Anan Jarab, Alaa Hammad, Fawaz Al-Asmari, Rahaf Zidan, Judith Eberhardt

**Affiliations:** ^1^Department of Pharmacy, Faculty of Pharmacy, Al-Zaytoonah University of Jordan, Amman, Jordan; ^2^College of Pharmacy, Al Ain University, Abu Dhabi, United Arab Emirates; ^3^Department of Clinical Pharmacy, Faculty of Pharmacy, Jordan University of Science and Technology, Irbid, Jordan; ^4^Department of Pharmacology and Toxicology, College of Pharmacy, King Saud University, Riyadh, Saudi Arabia; ^5^Department of Psychology, School of Social Sciences, Humanities and Law, Teesside University, Middlesbrough, United Kingdom

**Keywords:** eHealth, health literacy, validation, Confirmatory Factor Analysis, Rasch analysis, cultural contexts

## Abstract

**Introduction:**

Amidst the rapid digitalization of healthcare, there is a need for tools that accurately assess eHealth literacy across cultural contexts. This study focused on the validation of an Arabic version of the eHealth Literacy Questionnaire (eHLQ), a tool to facilitate patient engagement and health outcomes in digital healthcare.

**Method:**

Using a convenience sampling method, the study recruited a diverse sample of 657 participants from Jordan (58.9% females and 41.1% males).

**Results:**

Confirmatory Factor Analysis and Rasch analysis supported a six-factor model and demonstrated satisfactory item performance within established thresholds. The findings revealed good internal consistency with Cronbach’s α ranging between ranging between 0.71 to 0.84. Differential Item Functioning analysis indicated no gender-specific variations.

**Discussion:**

The validated Arabic eHLQ is a reliable tool that can help in supporting the development of tailored interventions to improve healthcare delivery in Arabic-speaking regions.

## 1 Introduction

Health literacy involves understanding and interpreting health information to make health-related decisions (1). Education, health awareness, and the skills needed to read and interpret medicine bottles, appointment slips, transit cards, and doctor’s orders are all part of health literacy. It equips individuals with the ability to navigate the complex world of health care and disease management (2). Health literacy is essential for accessing and using healthcare. It enables individuals to make informed choices by understanding health issues, diseases, and treatment options (1).

Healthcare systems face the challenge of managing an increasing volume of healthcare-related information and clinical records (3). Simultaneously, evolving information technology offers solutions by enabling the management of vast amounts of information through computerized storage of health records (4, 5). The advent of computer technologies has prompted healthcare officials to prioritize their integration within the healthcare system. This initiative has proven effective in sectors such as laboratories and pharmacies (6). Norman and Skinner (7) proposed the concept of e-health literacy, which refers to the capacity to effectively access, locate, and evaluate health-related information from electronic sources in order to address health-related issues. E-health literacy encompasses six fundamental competencies: traditional literacy, which includes reading, understanding, communicating, and writing; health literacy, focused on accessing, comprehending, evaluating, and applying health-related information; information literacy, which entails the effective access and use of information; media literacy, involving the ability to select, understand, evaluate, and create media-based messages; scientific literacy, which uses scientific methods to understand, evaluate, and explain health situations; and computer literacy, particularly in troubleshooting computer issues. Successful use of eHealth resources requires individuals to possess digital health literacy skills.

Low health literacy has been linked to poorer health outcomes and increased healthcare disparities, which makes it an essential area of focus in public health interventions (8). With the increasing reliance on digital health platforms, individuals without adequate eHealth literacy may face additional barriers to accessing healthcare and using digital resources effectively (9).

Assessing eHealth literacy is essential for understanding the use of eHealth platforms and resources. One of the first tools that was developed to assess health literacy was the eHealth Literacy Scale (eHEALS) (9), which remains a widely used tool that was applied to evaluate eHealth literacy and its impact on different health outcomes in different settings and populations (10, 11). However, the connections between these studies’ findings and specific eHealth recommendations were generally unclear (10). Furthermore, as noted by eHEALS author (12), the digital environment had substantially evolved since 2006, especially in terms of interactivity and information and communication technologies (ICT) capabilities and suggested revising the eHealth Literacy concept and eHEALS. Moreover, the studies that evaluated the construct of the eHEALS produced inconsistent results related to the number of factors and the distribution of the items between the different factors (13–19).

To overcome these drawbacks the eHealth Literacy Questionnaire (eHLQ) (20) was developed. The tool is a 35-item measure comprising seven domains of eHealth literacy. It measures various domains of eHealth literacy such as using technology to process health information, understanding electronic health information, finding reliable electronic health information, engaging with digital health services, feeling secure when using eHealth resources, and being motivated to engage with digital health. While this questionnaire has been validated in English, Danish, and Norwegian populations, no equivalent tool currently exists for Arabic-speaking populations, despite the significant number of Arabic speakers globally (21) and their growing engagement with digital health platforms (22).

The present study aimed to validate, assess for trustworthiness, and test the stability of the Arabic eHLQ among Jordanian adults. Jordan, with its high internet penetration rate and increasing reliance on digital health platforms (23), provided an ideal setting for this study. By evaluating Arabic speakers’ electronic health literacy with a validated and culturally appropriate questionnaire, the study aimed to aid healthcare providers and researchers in designing targeted interventions to improve health literacy and health outcomes.

## 2 Materials and methods

This cross-sectional study collected data using both paper-based and electronic questionnaires. Data was collected at a single time point from participants. Ethical approval was obtained from Al-Zaytoonah University of Jordan (Ref no:03/2023-2024), and the study adhered to the Declaration of Helsinki’s ethical principles for medical research involving human subjects.

### 2.1 Sample and sampling method

This study employed a convenience sampling method. A participant-to-item ratio of 10:1 was used to obtain the required sample size for factor analysis (24). The study targeted all citizens residing in Jordan as its population. To ensure geographical representation, participants were recruited from various central regions across the country. Inclusion criteria required participants to be Jordanian citizens aged 18 years or older, and literate in reading and writing. Exclusion criteria stipulated that individuals under the age of 18 years and those residing outside Jordan were ineligible to participate in the study. The recruiters were instructed to approach individuals from different age groups and from different sociodemographic statuses.

A total of 657 participants completed the questionnaire. Paper and electronic data collection method was adopted, with approximately one-third of surveys distributed in paper format and the remainder electronically. Distribution channels included community centers, healthcare facilities, and online platforms. Both the online and paper questionnaires included an introductory paragraph that clarified the study’s aim, participants’ rights and roles, inclusion and exclusion criteria, and the consent form.

### 2.2 Instrument

The survey instrument, consisting of 45 questions, gathered both demographic details and health literacy insights. The demographic data contained ten items to collect data about key sociodemographic aspects, including age, gender, monthly income, presence of chronic illnesses, self-assessed health status, education level, involvement in the medical field (study or work), preferred methods for accessing medical information online, and duration of daily online activity.

The second part of the survey consisted of the Arabic version of eHLQ (20), developed in accordance with the eHealth Literacy Framework (eHLF) proposed by Norgaard et al. (25). This self-report measure is composed of seven dimensions with a total of 35 items: five items for each of the first five dimensions, six items for the sixth dimension, and four items for the seventh dimension. The scale uses an ordinal response format, with responses graded on a Likert scale ranging from 0 (strongly disagree) to 3 (strongly agree). It comprises seven dimensions: 1- using technology to process health information, 2- understanding health concepts and language, 3- ability to actively engage with digital services, 4- feeling safe and in control, 5- motivated to engage, 6- access to working digital services, and 7- digital services that meet individual needs (20).

### 2.3 Tool validation

The eHLQ was chosen by an expert panel composed of two clinical pharmacists and one public health specialist. The selection was based on the questionnaire’s comprehensive coverage of various e-health literacy domains and its use of simplified language. The content validity of the questionnaire was confirmed by the expert panel and by members of the general population. The survey was translated into Modern Standard Arabic following the Brislin principle (26) to ensure that the Arabic version retained the original meaning of the questionnaire with cultural relevance. The forward-backward translation process was conducted by separate independent translators. The translated versions were compared, and a final Arabic version was produced. Thirty participants were recruited for a pilot study to assess the questionnaire’s face validity. Participants were randomly selected and briefed on the study’s purpose. They were asked to complete the questionnaire and participate in an open discussion to provide feedback. Specifically, they were asked to evaluate the relevance, clarity, content and simplicity of the items. Ultimately, the participants confirmed the adequacy of the eHLQ as all the participants found it easy to comprehend and complete, with no further modifications being necessary.

With the advancement of psychometric methods adopted to validate different health tools, the selection of the most adequate method may be demanding (27). The present paper applied Rasch model theory and classical test theory (CTT) to increase the validity and reliability of the study results.

### 2.4 Data analysis

The Statistical Package for the Social Sciences software (SPSS) version 23 (28) and RStudio Software (29) with packages TAM version 4.2–21 (30) and lavaan version 0.6–17 (31) were used for data analysis. All continuous variables were expressed as medians and interquartile ranges. For categorical variables, frequencies and percentages were reported. The internal consistency of each scale was evaluated by computing Cronbach’s alpha and McDonald’s ωt with acceptable values >0.7 (27, 32).

Confirmatory Factor Analysis (CFA) for ordinal data Diagonally Weighted Least Squares (DWLS) estimator was conducted to verify the fitness of the original 7-factor model, and scaled model fit indices, including Comparative Fit Index (CFI), Tucker-Lewis Index (TLI), Root Mean Square Error of Approximation (RSMEA), Standardized Root Mean Square Residual (SRMR), chi-square with degrees of freedom and *p*-value, and Minimum Discrepancy of Confirmatory Factor Analysis/Degrees of Freedom (CMIN/DF), were computed. The acceptable values were as follows: for CMIN/DF < 5 (33), for RMSEA <0.08 (34), for SRMR ≤0.08 (35) and for TLI and CFI values closer to 1 (36).

Multidimensional Rasch analysis was performed, and item thresholds were examined. Disordered thresholds may indicate irregularity and may arise when respondents fail to select the appropriate response options, which can result from unclear labeling or an excessive number of answer options. Model fit was assessed by computing item/person separation reliability. Item infit and outfit mean square values (MNSQs) were assessed, with acceptable range set between 0.5 and 1.5 (37). Additionally, differential item functioning (DIF) was evaluated to examine potential biases resulting from gender differences with acceptable logit differences of ≥0.43 (38). Moreover, ceiling or floor effects were evaluated by computing the frequency of participants who scored the maximum and minimum possible scores.

## 3 Results

Table 1 presents the sociodemographic profile of the study participants. A total of 657 individuals took part. The median age was 26 years, with ages ranging from 22 to 31 years. Regarding gender distribution, 58.9% of participants identified as female and 41.1% as male. In terms of marital status, the majority (62.3%) reported being single, with the remaining 37.7% being married. Regarding income status, 51% reported earning less than 500 Jordanian Dinars (JOD) per month, 35% reported a monthly income between 500 and 1,000 JOD, and 14% earned more than 1,000 JOD per month. Concerning health, 11.4% of participants reported having a chronic disease, while 88.6% indicated no chronic conditions. Additionally, 42.8% of participants reported that at least one family member had a chronic disease, compared to 57.2% who reported no family history of chronic illness.

**Table 1:** Sociodemographic profile of the participants (*n* = 657).

Variables		Median (25–75) or frequency (%)
Age		26 (22–31)
Gender	Female	387 (58.9%)
	Male	270 (41.1%)
Marital status	Single	409 (62.3%)
	Married	248 (37.7%)
Monthly income	Less than 500JOD	335 (51%)
	500–1000JOD	230 (35%)
	More than 1000JOD	92 (14%)
Do you have any chronic diseases?	No	582 (88.6%)
	Yes	75 (11.4%)
Does any of your family members have any chronic diseases?	No	376 (57.2%)
	Yes	281 (42.8%)

**Figure 1:**
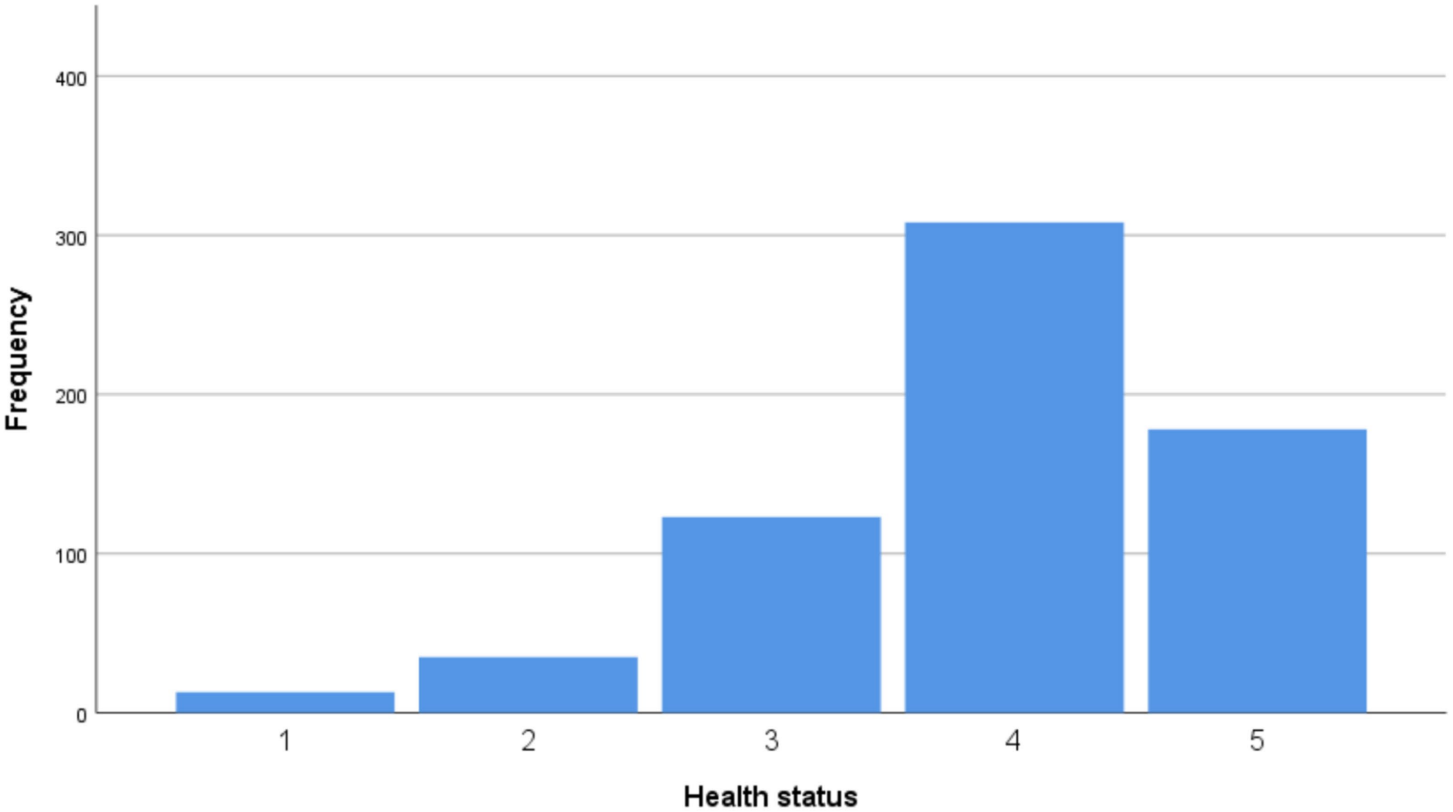
Evaluation of health status from 1 (low) to 5 (high).

The participants’ perceptions of their current health status are depicted in Figure 1. The majority of participants rated their health status as 4 out of 5 (46.9%), followed by a rating of 5 out of 5 (27.1%).

**Figure 2:**
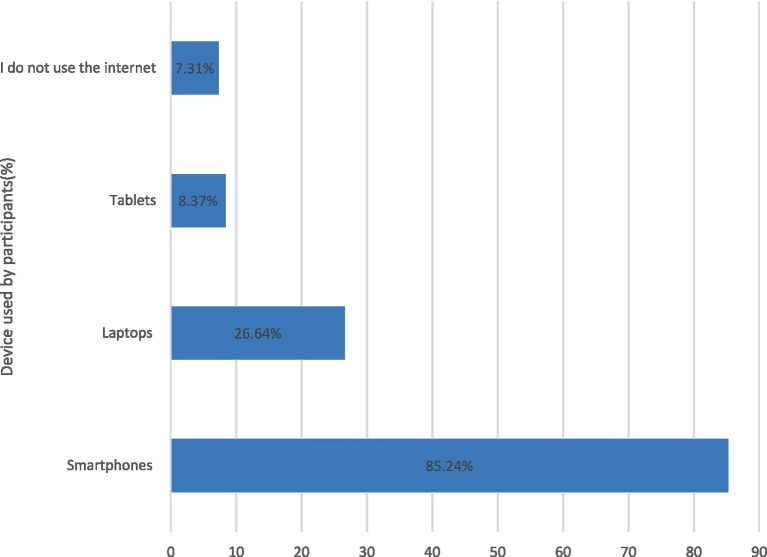
Devices used to access medical information online.

Devices that participants used to access medical information online are presented in Figure 2. Most participants used smartphones (85.23%), followed by laptops (26.63%), with tablets being the least used (8.37%). Additionally, 7.30% of participants reported that they did not use the Internet to access medical information.

The evaluation of participants’ eHealth Literacy Questionnaire responses is summarized in Table 2. Most participants agreed or strongly agreed with Q6 (94%), followed by Q4 (92.5%). Conversely, most disagreed or strongly disagreed with Q3 (49%), followed by Q20 (46.6%). All items had a median score of 2.

**Table 2:** Frequency distribution of participants’ responses to eHLQ items, with quantiles.

Q number	Strongly disagree	Disagree	Agree	Strongly agree	Median (25–75 percentiles)
1. Using technology to process health information					
Q7	3 (0.5%)	52 (7.9%)	354 (53.9%)	248 (37.7%)	2 (2–3)
Q11	10 (1.5%)	123 (18.7%)	350 (53.3%)	174 (26.5%)	2 (2–3)
Q13	9 (1.4%)	106 (16.1%)	402 (61.2%)	140 (21.3%)	2 (2–2)
Q20	33 (5%)	273 (41.6%)	280 (42.6%)	71 (10.8%)	2 (1–2)
Q25	23 (3.5%)	239 (36.4%)	309 (47%)	86 (13.1%)	2 (1–2)
2. Understanding of health concepts and language					
Q5	11 (1.7%)	66 (10%)	362 (55.1%)	218 (33.2%)	2 (2–3)
Q12	11 (1.7%)	109 (16.6%)	379 (57.7%)	158 (24%)	2 (2–2)
Q15	28 (4.3%)	133 (20.2%)	344 (52.4%)	152 (23.1%)	2 (2–2)
Q21	6 (0.9%)	78 (11.9%)	398 (60.6%)	175 (26.6%)	2 (2–3)
Q26	8 (1.2%)	91 (13.9%)	409 (62.3%)	149 (22.7%)	2 (2–2)
3. Ability to actively engage with digital services					
Q4	9 (1.4%)	40 (6.1%)	288 (43.8%)	320 (48.7%)	2 (2–3)
Q6	2 (0.3%)	37 (5.6%)	313 (47.6%)	305 (46.4%)	2 (2–3)
Q8	31 (4.7%)	201 (30.6%)	307 (46.7%)	118 (18%)	2 (1–2)
Q17	9 (1.4%)	112 (17%)	389 (59.2%)	147 (22.4%)	2 (2–2)
Q32	12 (1.8%)	93 (14.2%)	425 (64.7%)	127 (19.3%)	2 (2–2)
4. Feeling safe and in control					
Q1	12 (1.8%)	52 (7.9%)	335 (51%)	258 (39.3%)	2 (2–3)
Q10	12 (1.8%)	140 (21.3%)	374 (56.9%)	131 (19.9%)	2 (2–2)
Q14	12 (1.8%)	163 (24.8%)	376 (57.2%)	106 (16.1%)	2 (1–2)
Q22	15 (2.3%)	182 (27.7%)	365 (55.6%)	95 (14.5%)	2 (1–2)
Q30	13 (2%)	122 (18.6%)	403 (61.3%)	119 (18.1%)	2 (2–2)
5. Motivated to engage with digital services					
Q2	13 (2%)	125 (19%)	388 (59.1%)	131 (19.9%)	2 (2–2)
Q19	7 (1.1%)	108 (16.4%)	388 (59.1%)	154 (23.4%)	2 (2–2)
Q24	34 (5.2%)	225 (34.2%)	307 (46.7%)	91 (13.9%)	2 (1–2)
Q27	8 (1.2%)	105 (16%)	417 (63.5%)	127 (19.3%)	2 (2–2)
Q35	14 (2.1%)	84 (12.8%)	362 (55.1%)	197 (30%)	2 (2–3)
6. Access to digital services that work					
Q3	64 (9.7%)	258 (39.3%)	240 (36.5%)	95 (14.5%)	2 (1–2)
Q9	36 (5.5%)	192 (29.2%)	337 (51.3%)	92 (14%)	2 (1–2)
Q16	38 (5.8%)	235 (35.8%)	273 (41.6%)	111 (16.9%)	2 (1–2)
Q23	23 (3.5%)	280 (42.6%)	291 (44.3%)	63 (9.6%)	2 (1–2)
Q29	22 (3.3%)	190 (28.9%)	343 (52.2%)	102 (15.5%)	2 (1–2)
Q34	10 (1.5%)	136 (20.7%)	390 (59.4%)	121 (18.4%)	2 (2–2)
7. Digital services that suit individual needs					
Q18	9 (1.4%)	157 (23.9%)	385 (58.6%)	106 (16.1%)	2 (1–2)
Q28	17 (2.6%)	177 (26.9%)	366 (55.7%)	97 (14.8%)	2 (1–2)
Q31	19 (2.9%)	202 (30.7%)	364 (55.4%)	72 (11%)	2 (1–2)
Q33	8 (1.2%)	108 (16.4%)	405 (61.6%)	136 (20.7%)	2 (2–2)

### 3.1 Scale 1: using technology to process health information

Most participants indicated agreement or strong agreement across items, with Q7, Q11, and Q13 showing high levels of agreement (median = 2). However, items Q20 and Q25 had notable proportions of disagreement, suggesting variability in ease of use.

### 3.2 Scale 2: understanding of health concepts and language

Participants generally agreed with items assessing health concept comprehension, as seen in Q5 and Q12 (median = 2), although some disagreement was observed for Q15 (24.5%).

### 3.3 Scale 3: ability to actively engage with digital services

High levels of agreement were reported, particularly for items like Q4 and Q6 (median = 2). Q8 showed a slightly higher disagreement rate (30.6%), which indicated some variability in engagement levels.

### 3.4 Scale 4: feeling safe and in control

Most participants felt safe and in control when using digital health services, with items Q1 and Q10 reflecting strong agreement (median = 2). However, Q14 and Q22 had higher disagreement levels, which suggested some concerns around safety.

### 3.5 Scale 5: motivated to engage with digital services

The majority agreed with items assessing motivation, such as Q2 and Q19 (median = 2), although Q24 showed more disagreement (34.2%), which indicated mixed motivation levels.

### 3.6 Scale 6: access to digital services that work

Responses varied, with some participants finding digital services functional and accessible, while others faced usability issues, as reflected in Q3 and Q16 (median = 2).

### 3.7 Scale 7: digital services that suit individual needs

There was variability in whether services met individual needs. For example, Q18 and Q28 showed high agreement, though Q28 and Q31 had disagreement proportions around 27–30%.

Overall, none of the participants recorded the minimum score of 35, and only 1% achieved the maximum score of 140, indicating the absence of ceiling and floor effects. All items had a median score of 2, showing a general tendency toward agreement.

### 3.8 Tool validation

CFA was conducted to evaluate the 7-factor model suggested in the original questionnaire. However, the covariance matrix of latent variables was not positive definite due to excessive correlation between factors 6 and 7, which prevented model convergence. Consequently, these two factors were combined into a single factor, and a new analysis was conducted using a 6-factor model.

Cronbach’s alphas and McDonald’s ωt of the 6 factors are presented in Table 3. Across all scales, the reliability coefficients were notably high, affirming the internal consistency of the eHLQ and supporting its validity for assessing various aspects of engagement with digital health services. Specifically, all scales exhibited reliability coefficients above 0.7, ranging between 0.71 for Factor 3 to 0.84 for factor 6, confirming their adequacy for research use.

**Table 3:** Internal consistency of the Arabic version of the eHLQ.

Scale	Cronbach’s alpha	McDonald’s ωt
1. Using technology to process health information	0.74	0.75
2. Understanding of health concepts and language	0.73	0.73
3. Ability to actively engage with digital services	0.71	0.71
4. Feel safe and in control	0.76	0.76
5. Motivated to engage with digital services	0.74	0.74
6. Access to digital services that work & digital services that suit individual needs	0.86	0.86
eHLQ	0.94	0.94

The chi-square test for the 6-factor model was significant (chi-square = 2.032, df = 545, *p* < 0.001) indicating inadequacy of the model. However, this was expected due to the large sample size; therefore, the method of dividing chi-square by degree of freedom was applied and yielded acceptable CMIN/DF = 3.72. Other scaled model fit indices were also acceptable including RMSEA = 0.064 (90Cl: 0.062–0.068), SRMR = 0.063, CFI = 0.93, and TLI = 0.92. Standardized factor loadings ranged from 0.42 to 0.81. The highest factor loadings were observed for Q14 and Q22 in Factor 4, while the lowest were recorded for Q3 in Factor 6 and only two items were < 0.5. Detailed standardized factor loadings for each item are provided in Table 4.

**Table 4:** Reliability, factor loadings, outfit and infit statistics, item locations, extraction rates, and Andrich-Rasch thresholds of the eHLQ.

Item	Standardized factors loadings (SE)	Outfit MNSQ	Infit MNSQ	Location	Tau parameters		
					1	2	3
1. Using technology to process health information							
Q7	0.64 (1)	1.02	1.01	−2.34	−4.84	−2.77	0.6
Q11	0.63 (0.06)	1.01	1.02	−1.53	−4.17	−1.68	1.26
Q13	0.74 (0.07)	0.88	0.89	−1.48	−4.18	−1.92	1.67
Q20	0.67 (0.07)	1.08	1.08	−0.32	−3.32	−0.22	2.58
Q25	0.76 (0.07)	0.94	0.94	−0.63	−3.65	−0.56	2.34
2. Understanding of health concepts and language							
Q5	0.63 (1)	1.06	1.03	−1.77	−3.84	−2.36	0.88
Q12	0.71 (0.07)	0.94	0.96	−1.49	−4.08	−1.87	1.48
Q15	0.6 (0.06)	1.14	1.12	−1.04	−3.22	−1.41	1.52
Q21	0.68 (0.07)	0.98	0.98	−1.84	−4.49	−2.35	1.32
Q26	0.74 (0.07)	1.03	1.02	−1.61	−4.29	−2.15	1.61
3. Ability to actively engage with digital services							
Q4	0.6 (0)	1.06	1.01	−2.03	−3.57	−2.54	0
Q6	0.62 (0.08)	0.94	0.99	−2.55	−4.82	−2.96	0.13
Q8	0.65 (0.08)	1.05	1.04	−0.7	−3.12	−0.74	1.78
Q17	0.77 (0.09)	0.91	0.91	−1.43	−4.04	−1.76	1.53
Q32	0.69 (0.08)	1.01	1.01	−1.27	−3.67	−1.94	1.79
4. Feeling safe and in control							
Q1	0.46 (0)	1.36	1.25	−1.91	−3.73	−2.53	0.53
Q10	0.7 (0.15)	0.96	0.97	−1.3	−4.17	−1.56	1.82
Q14	0.81 (0.16)	0.88	0.89	−1.14	−4.25	−1.34	2.17
Q22	0.79 (0.17)	0.9	0.9	−0.96	−4.07	−1.14	2.33
Q30	0.72 (0.15)	0.95	0.97	−1.25	−4.01	−1.74	2
5. Motivated to engage with digital services							
Q2	0.61 (0)	1.07	1.06	−1.26	−3.89	−1.66	1.78
Q19	0.72 (0.06)	0.89	0.9	−1.61	−4.43	−1.92	1.51
Q24	0.62 (0.07)	1.1	1.11	−0.5	−3.19	−0.59	2.27
Q27	0.74 (0.07)	0.94	0.95	−1.46	−4.28	−1.96	1.86
Q35	0.68 (0.07)	0.98	0.98	−1.54	−3.63	−2.04	1.05
6. Access to digital services and digital services that suit individual needs							
Q3	0.42 (1)	1.58	1.52	−0.17	−3.01	−0.83	2.26
Q9	0.6 (0.15)	1.17	1.15	−0.53	−3.06	−0.46	1.89
Q16	0.7 (0.16)	0.98	0.98	−0.54	−3.69	−0.24	2.75
Q23	0.68 (0.15)	0.97	0.97	−0.39	−3.53	−0.96	2.11
Q29	0.74 (0.16)	0.9	0.91	−0.8	−4.16	−1.57	1.88
Q34	0.74 (0.17)	0.88	0.9	−1.28	−4.35	−1.39	2.09
Q18	0.73 (0.17)	0.98	0.98	−1.22	−3.76	−1.13	2.21
Q28	0.77 (0.17)	0.83	0.84	−0.89	−3.71	−0.91	2.65
Q31	0.74 (0.16)	0.88	0.89	−0.66	−4.25	−1.89	1.71
Q33	0.74 (0.17)	0.91	0.92	−1.48	−4.84	−2.77	0.6

### 3.9 Rasch model

A six-dimensional model was analyzed. The person reliability index for the 6 dimensions ranged between 0.68 for dimension 3 to 0.86 for dimension 6, and the item separation reliability ranged between 0.85 for dimension 3 to 0.91 for dimension 6. Table 4 displays the infit and Outfit mean square values, affirming the eHLQ’s ability to differentiate between various participant levels and confirming the model’s item hierarchy. Notably, the only item slightly exceeding the acceptable range was Q3, with an Outfit MNSQ of 1.58 and an infit MNSQ of 1.52. All questions were presented with ordered response categories. Analysis revealed that Q3 was the most challenging item for participants to answer, followed by Q20, whereas Q6 was the most straightforward. Furthermore, the easiest threshold to respond to was the first threshold of Q7, followed by Q6. Conversely, the third threshold of Q23 was identified as the most challenging. Scale 6 recorded the highest outfit and infit values for Q3, at 1.58 and 1.52, respectively. Following this, Scale 4 for Q1 showed values of 1.36 for outfit and 1.26 for infit. The lowest values were observed on Scale 6 for Q28, with outfit and infit values of 0.83 and 0.84, respectively.

**Figure 3:**
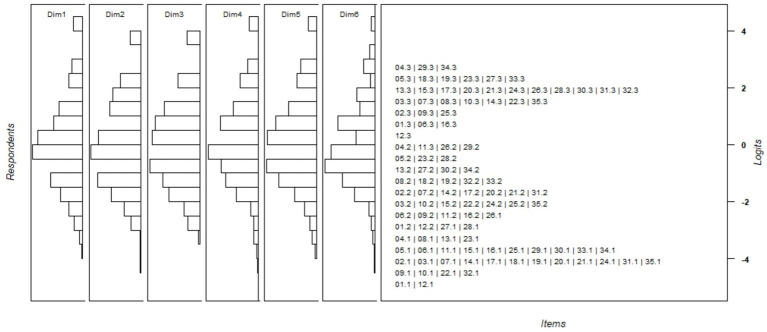
Wright map of the Rasch analysis. The left panel displays the ability level area of the respondents for both factors, while the right panel shows the item difficulty level area.

DIF between genders was evaluated and the analysis revealed that the difference between the two genders on the logit scale was 0.16 logits, indicating no significant differences in DIFs between the two sexes as the recommended cutoff point is ≥0.43. Figure 3 displays the Wright map, showing that participants were distributed across all difficulty levels in all 6 domains, with the majority concentrated in the middle range. The item thresholds revealed a range of item difficulties, all with well-ordered thresholds.

## 4 Discussion

The current study focused on translating and validating the 35-item eHLQ into Arabic, aiming to facilitate its cross-cultural adaptation for researchers in Arabic-speaking healthcare contexts. The Arabic version of the eHLQ showed strong internal consistency, with acceptable alpha coefficients in all six domains. The results of this study are consistent with those from eHLQ validation studies conducted in other languages (39, 40) confirming the robustness of the questionnaire across different cultural and linguistic settings. In comparison, the reliability of the Arabic version of the eHLQ is on par with, or slightly exceeds, that of its counterparts in other languages. This finding reinforces its utility and robustness in Arabic-speaking healthcare contexts.

Our CFA supported a 6-factor model, which has a different number of factors to the original 7-factor model (20). This adjustment was due to a high correlation between the factors “Access to digital services” and “Digital services that suit individual needs,” which resulted in their combination into a single factor. The merging of these two factors can be theoretically justified, as both assess access to digital services with similar items, such as “eHealth systems provide me with easy ways to get what I need” from the original factor “Digital services that suit individual needs” and “I have access to health technology that works” from the original factor “Access to digital services.” Nevertheless, certain items, such as items 1 and 3, exhibited relatively lower factor loadings. These discrepancies suggest potential challenges in translation or variations in respondents’ perceptions, possibly influenced by demographic characteristics. These findings highlight the importance of further examining these discrepancies to improve the questionnaire’s validity and applicability in diverse cultural settings. Such an investigation could help identify specific cultural or linguistic factors that influence response patterns, thus enhancing the tool’s effectiveness for global health assessments. Addressing these challenges in future iterations may help to refine the tool further and enhance its cross-cultural applicability.

Utilizing Rasch analysis to assess the proposed model fit, most items demonstrated satisfactory fit within acceptable thresholds, except for Item 3 which exhibited a slightly high infit/outfit value, indicating potential under-discrimination. It is important to note that outfit statistics are particularly sensitive to responses to items whose difficulty differs significantly from an individual’s ability level while infit statistics is more sensitive to the pattern of responses to items targeted on the person (41, 42).

This study revealed a pronounced ease of interaction with digital tasks. The thresholds were consistently reasonably low, reflecting ease of engagement with digital health information and motivation to use digital services. Overall, while previous studies identified basic digital tasks as relatively easy, our study highlights a greater comfort and proficiency in using digital health platforms among respondents. Conversely, items Q20, Q5, Q4, Q1, Q35, Q23, and Q31, which were distributed across all the questionnaire domains, were identified as the most challenging, indicating that individuals with physical ailments may struggle to access information on managing mental health issues. These observed variations in participants’ health literacy levels are consistent with the understanding that health literacy encompasses not just knowledge, but also the ability to perform responsibilities and tasks related to health and healthcare effectively. This suggests that health literacy involves a dynamic set of skills that vary widely among individuals, impacting their ability to manage their health and interact with healthcare systems.

Consistent with previous validations of the eHLQ in other languages, the Arabic version exhibited strong reliability and validity. The overall internal consistency (Cronbach’s *α* = 0.71–0.86) is comparable to or slightly higher than the reliability coefficients reported in Danish (43)(*α* = 0.75–0.87) and Norwegian populations (*α* = 0.73–0.90) (20). Similar to these studies, the Arabic eHLQ demonstrated strong performance across multiple domains, particularly in assessing access to digital services and users’ motivation to engage with digital platforms. The merging of Factors 6 and 7 in this study aligns with findings from a prior validation, which reported high correlations between these domains (20). Additionally, certain items (e.g., Q3 and Q20) exhibited higher variability in response patterns, consistent with earlier research highlighting these items as universally challenging across diverse populations. These results reinforce the robustness of the eHLQ framework while emphasizing the importance of contextual adaptations to address subtle cultural differences in item interpretation.

The findings of the present study underscore the robust validity and reliability of the Arabic version of the eHLQ developed in this research, and evidence the proficiency of Jordanian participants in utilizing digital health services, consistent with trends in other cultural contexts. They also have practical implications for healthcare providers, policymakers, and researchers. The validated Arabic eHLQ offers a reliable tool to assess eHealth literacy, enabling the design of targeted interventions to improve digital health engagement in Arabic-speaking populations. For instance, the tool can be used to identify individuals or groups who may benefit from tailored educational programs or digital skill training, thereby promoting equitable access to healthcare resources.

### 4.1 Strengths, limitations, and future directions

One of the primary strengths of this study is the large sample size, which contributes significantly to the reliability and validity of our findings. A larger sample size helps mitigate random fluctuations and reduce sampling errors, thereby increasing confidence in the study’s results. Such reliability is essential for producing dependable research outcomes that can inform further studies and practical applications.

Furthermore, the substantial sample size facilitated detailed subgroup analyses, which enabled the exploration of nuances and variations among demographic and other relevant factors. This deeper exploration allowed for more insightful conclusions and the identification of important insights that might otherwise be missed.

However, despite these strengths, the study is not without its limitations. Convenience sampling techniques were applied which may be susceptible to selection bias, as certain individuals might be more inclined to participate than others, potentially skewing the results. Nevertheless, the young sample mimics the young Jordanian population, which has a median age of 22.4 years according to the Jordanian High Population Council (44). Moreover, like the present study sample, more than 41% of Jordanian population has a monthly household of less than 500 JDs (45). Furthermore, the study’s aim was to validate the tool within the general population. Additionally, with this being a study based on self-report, it may have been left susceptible to recall and social desirability biases, where participants may not have accurately remembered past events or may have responded in a manner they perceived as favorable rather than truthful. Although the present study went through a systemic translation process, semantic bias cannot be completely excluded.

Future research could benefit from employing a more stratified sampling technique to minimize selection bias and ensure a more representative cross-section of the population, including targeting special populations such as the older adult, patients of different chronic diseases, low education levels and rural residents. Additionally, to mitigate the effects of recall and social desirability biases inherent in self-report studies, future investigations could implement mixed methods approaches that include qualitative interviews could also provide deeper insights into the motivations behind participants’ responses, offering a more nuanced understanding of their health literacy and digital engagement. Finally, although this study applied rigorous validation methodology it did not evaluate test-rest reliability which could be conducted in future research.

## 5 Conclusion

This study provides a comprehensive development and evaluation of the Arabic version of the eHLQ, focusing on its accuracy, reliability, and applicability within Arabic-speaking populations. The findings demonstrate that the Arabic translation of the eHLQ maintains a high level of internal consistency, comparable to or surpassing the reliability of versions in other languages. CFA and Rasch analysis both supported the tool’s effectiveness, with the single-factor model fitting adequately and most items performing well within acceptable thresholds. With the six-factor model demonstrating adequate fit and most items performing well within acceptable thresholds. With this validation, the questionnaire can now be used to assess the e-health literacy of Arabic-speaking populations. This is crucial for enhancing health outcomes and facilitating greater patient involvement in digital healthcare settings.

The study’s findings provide useful insights for healthcare policymakers and practitioners aiming to improve digital health practices in Jordan and other Arabic-speaking countries. By utilizing a culturally and linguistically customized eHLQ, health workers can gain a deeper understanding of patients’ e-health literacy. This understanding, in turn, facilitates the enhancement of digital health resource utilization among patients in these countries. Such targeted improvements in e-health literacy can promote more effective and inclusive digital healthcare services, ultimately fostering better health outcomes.

## Data Availability

The raw data supporting the conclusions of this article will be made available by the authors, without undue reservation.
